# Genome-wide association study reveals the genetic determinism of serum biochemical indicators in ducks

**DOI:** 10.1186/s12864-022-09080-9

**Published:** 2022-12-27

**Authors:** Hehe Tang, He Zhang, Dapeng Liu, Zhen Wang, Daxin Yu, Wenlei Fan, Zhanbao Guo, Wei Huang, Shuisheng Hou, Zhengkui Zhou

**Affiliations:** 1 Key Laboratory of Animal (Poultry) Genetics Breeding and Reproduction, Ministry of Agriculture and Rural Affairs, State Key Laboratory of Animal Nutrition, Institute of Animal Science, Chinese Academy of Agricultural Sciences, Beijing, PR China; 2 College of Food Science and Engineering, Qingdao Agricultural University, Qingdao, PR China

**Keywords:** Serum, Biochemical indicators, GWAS, Duck

## Abstract

**Background:**

The serum is rich in nutrients and plays an essential role in electrolyte and acid–base balance, maintaining cellular homeostasis. In addition, serum parameters have been commonly used as essential biomarkers for clinical diagnosis. However, little is known about the genetic mechanism of the serum parameters in ducks.

**Results:**

This study measured 18 serum parameters in 320 samples of the F_2_ segregating population generated by Mallard × Pekin duck. The phenotypic correlations showed a high correlation between LDH, HBDH, AST, and ALT (0.59–0.99), and higher coefficients were also observed among TP, ALB, HDL-C, and CHO (0.46–0.87). And then, we performed the GWAS to reveal the genetic basis of the 18 serum biochemical parameters in ducks. Fourteen candidate protein-coding genes were identified with enzyme traits (AST, ALP, LDH, HBDH), and 3 protein-coding genes were associated with metabolism and protein-related serum parameters (UA, TG). Moreover, the expression levels of the above candidate protein-coding genes in different stages of breast muscle and different tissues were analyzed. Furthermore, the genes located within the high-LD region (r^2^ > 0.4 and − log_10_(*P*) < 4) neighboring the significant locus also remained. Finally, 86 putative protein-coding genes were used for GO and KEGG enrichment analysis, the enzyme-linked receptor protein signaling pathway and ErbB signaling pathway deserve further focus.

**Conclusions:**

The obtained results can contribute to new insights into blood metabolism and provide new genetic biomarkers for application in duck breeding programs.

**Supplementary Information:**

The online version contains supplementary material available at 10.1186/s12864-022-09080-9.

## Background

In the animal organism, blood components reflect immune activity and nutrient metabolism. The serum is the fluid and solute component of blood that does not contain leukocytes, erythrocytes, platelets, or clotting factors [1, 2]. Furthermore, the serum is rich in nutrients, including all proteins, electrolytes, antigens, antibodies, hormones, and exogenous substances not used in the clotting process. In addition, serum plays an essential role in electrolyte and acid–base balance, maintaining the homeostasis of the intracellular environment, and transporting nutrients to the body [3, 4]. Therefore, measuring the content of various serum components is helpful in many applications, such as medical diagnostics and animal husbandry.

Serum parameters are commonly used as essential biomarkers for clinical diagnosis in the medical field [5, 6]. For instance, serum Ca, phosphorus, and alkaline phosphatase (ALP) are essential indicators of bone metabolism [7]. The level imbalance of triglyceride (TG), cholesterol (CHO), high-density lipoprotein cholesterol (HDL-C), and low-density lipoprotein cholesterol (LDL-C) in serum were usually accompanied by lipid metabolism disease [8]. In animal breeding, serum biochemical parameters indirectly reflect animal health status and economic traits. Studies have found that high levels of Lactate Dehydrogenase (LDH), Creatine Kinase (CK), Blood glucose (GLU), and aspartate transaminase (AST) were associated with pale, soft, and exudative (PSE) meat. It provides a new and effective method for detecting PSE meat by measuring the blood biochemical parameters [9]. Dong compared serum biochemical parameters between two broiler chicken lines and identified serum HDL-C and LDL-C levels as potential biomarkers for selecting of lean birds [10]. It has been reported that comparing serum metabolite compositions between obese and lean-growing pigs based on the metabonomic approach provides a useful model for childhood obesity research [11]. In Shanma duck, it was reported that the possibility of early breeding of duck by using serum biomarkers [12]. In addition, the GWAS analysis of 42 days old Pekin duck found that 54 significant QTLs associated with 23 candidate genes may contribute to 12 serum parameters [13]. Therefore, serum biomarkers have been developed as indicators for clinical in humans and breeding purposes in animals, and elucidating the genetic basis of these serum biomarkers is critical to the livestock breeding process.

With the development of genome re-sequencing technology, more and more genome-wide association (GWAS) analyses have been performed on serum biochemical indicators of livestock and poultry in recent years. In different livestock and poultry, quantitative trait loci (QTL) for serum biochemical indicators have been identified [14–16]. Although studies have conducted GWAS analysis on serum parameters of poultry, there is a lack of GWAS focused on the early growth period of ducks. The study of serum parameters in the early stage of poultry growth can improve the efficiency of seed selection, conduct early assessment of poultry growth and development, and improve the economic benefits. At present, many studies are mainly limited to association analysis of serum parameters and phenotypic traits, the study of genetic variability in blood parameters could contribute to design new strategies to overcome the limited effectiveness of the traditional selection programs to improve disease resistance, tolerance and resilience [17]. In addition, serum parameters have been studied by GWAS in livestock and poultry [14, 18], but few studies have been conducted on their genetic basis in ducks. Hence, in this study, total 320 samples of 3-week-old ducks were used as experimental animals to perform the GWAS of serum parameters and identify the candidate regions and genes to facilitate early breeding.

## Results

### Phenotypic correlation between serum biochemical indicators

**Fig. 1:**
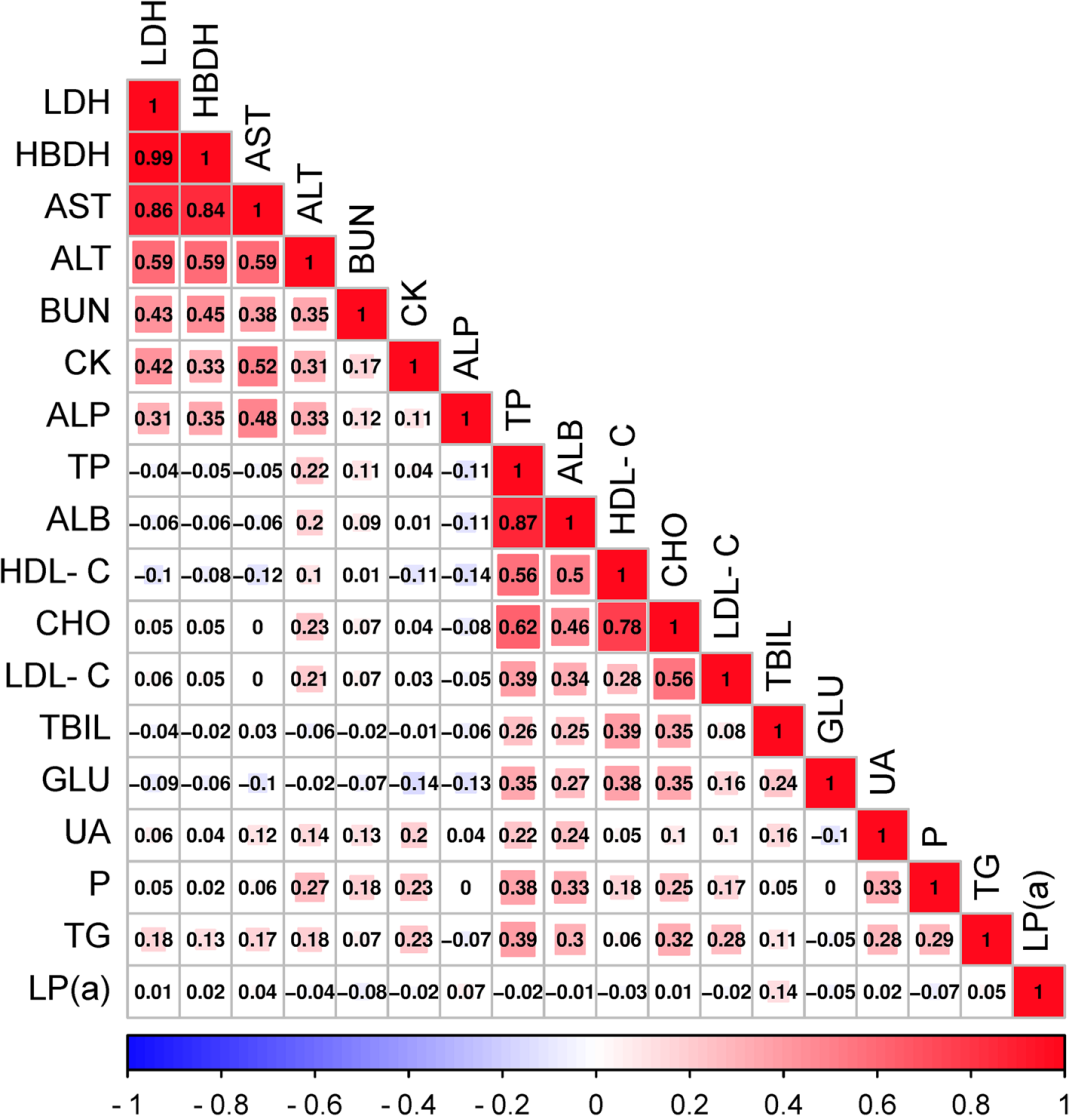
Pearson’s correlation coefficients among the 18 analyzed serum biochemical parameters. The value in the box represents the Pearson correlation coefficient between the two serum parameters

Eighteen serum biochemical parameters were detected in this study, including CHO, TG, ALP, UA, HDL-C, LDL-C, etc. By calculating the coefficient variation of all serum biochemical indicators in ducks, the TP (0.11), ALB (0.10), TBIL (0.16), GLU (0.11), P (0.11), CHO (0.13), and HDL-C (0.14) showed a lower variation coefficient. In contrast, some biochemical indicators have higher variation coefficients, mainly AST (0.59), ALP (1.22), (LP(a)) (0.55), LDH (0.54), and HBDH (0.76), the variation coefficients of which exceed more than 0.60 (Table S1). Through the correlation analysis of all 18 blood biochemical indicators, the Pearson correlation coefficient ranged from 0.46–0.87 among the serum indicators, including TP, ALB, HDL-C and CHO, and the higher correlation coefficients were observed among LDH, HBDH, AST, and ALT (*r* = 0.59–0.99) (Fig. 1).

### GWAS analysis

In this study, 18 serum biochemical indicators of 3-week-old F_2_ ducks, were selected as phenotypes for GWAS analysis (Correction threshold = 8.916). Significant signals in Manhattan plots were only observed among AST, ALP, LDH, HBDH, UA, and TG. The correlation QQ plots (Figure S1) showed that the model we used was reasonable, most of the observed *P* values were consistent with the expected values, and significant SNPs were found, indicating that the above association analysis results for serum parameter traits are reliable. The Manhattan and QQ plots for the other 14 serum biochemical indicators were indicated in Figure S2 and Figure S3, GWAS analysis revealed that these SNP were not significant.

The six biochemical indicators that showed significant signals above were divided into two categories: The enzyme traits (AST, ALP, LDH, and HBDH) and the metabolism and protein-related traits (UA and TG).

#### I. GWAS for enzyme traits

**Fig. 2:**
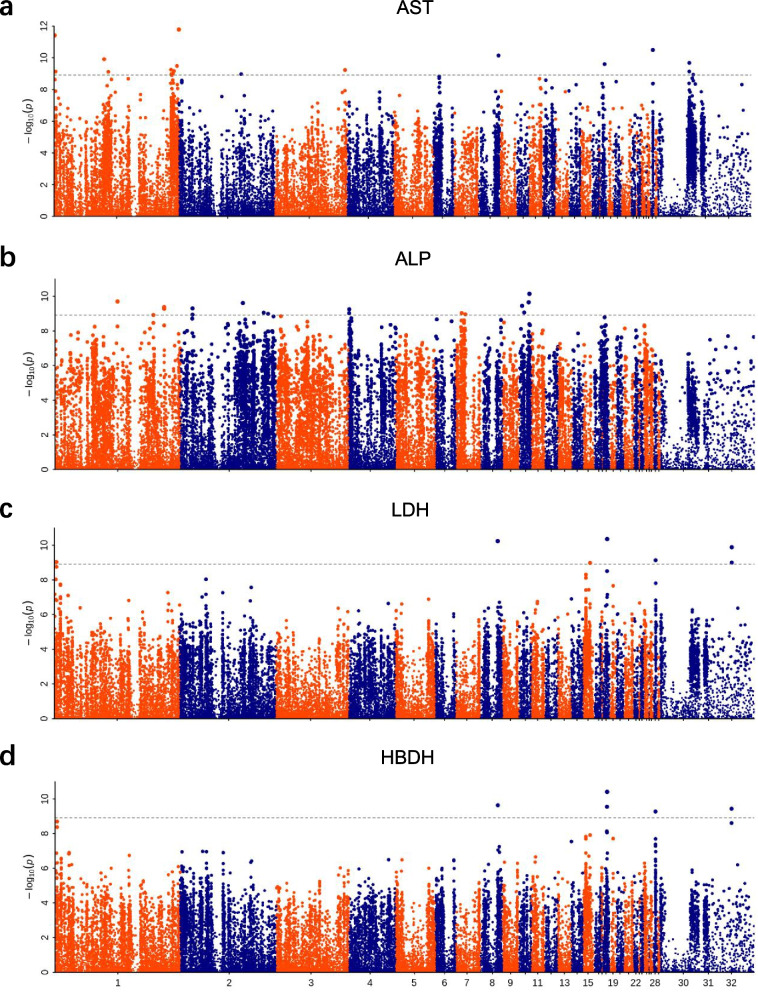
The Manhattan plots of the enzyme traits. **a** Manhattan plot of AST. **b** Manhattan plot of the ALP. **c** Manhattan plot of the LDH. **d** Manhattan plot of the HBDH. Abscissa numbers represent different chromosomes. The dotted line in the Figure represents the threshold level (Correction threshold = 8.916)

The Manhattan plot of AST showed obvious signals on chromosome 1 (Fig. 2a). A total of 20 SNPs reached the significant threshold level, of which 11 SNPs are distributed on chromosome 1. The genes annotated by Top10 SNPs are shown in Table 1, including *SHANK3*, *SPOP*, *SLC30A7*, *ABL1*, and *SLC26A5*. The results of ALP showed that 18 SNPs reached the significance threshold level (Fig. 2b), of which potential SNPs were mainly distributed in the *PCDH11X*, *LDLRAD4*, *ABCB7*, *CAPZA2*, *MALRD1*, and *PRSS12* (Table 1). Only 5 SNPs reached the significant threshold level regarding the results of LDH and were distributed in the *ABL1*, *NUP214*, and *KAT7* (Fig. 2c, Table 1)*.* Regarding HBDH, 7 SNPs reached the significant threshold level in the Manhattan plot (Fig. 2d), the genes annotated by these SNPs included *ABL1*, *KAT7*, *CAB2*, and *TRAP1* (Table 1).

**Table 1:** The top 10 single nucleotide polymorphisms (SNPs) identified in genome-wide association studies for the enzyme traits blood parameters

Trait	CHROM	POS	-log_10_(P)	REF	ALT	Close protein coding gene
AST	chr1	202770874	11.779	C	T	SHANK3
	chr1	870006	11.412	C	T	LOC113844826
	chr28	4089768	10.49	C	T	SPOP
	chr8	33250067	10.143	T	A	SLC30A7
	chr1	81119768	9.907	T	C	LOC110351494
	chr30	51699768	9.674	C	A	LOC101797811
	chr18	7483362	9.594	A	G	ABL1
	chr1	199642761	9.480	T	G	LOC113844094
	chr1	189958433	9.244	A	G	SLC26A5
	chr3	114552902	9.226	C	T	LOC110351724
ALP	chr10	16572239	10.140	A	G	PCDH11X
	chr1	101155580	9.700	C	T	LOC113842353-USP25
	chr10	15392758	9.655	C	T	LOC106016319
	chr2	101658067	9.612	G	A	LDLRAD4
	chr10	4639671	9.451	T	C	ABCB7
	chr1	176840631	9.378	T	C	CAPZA2
	chr2	19972003	9.303	C	T	MALRD1
	chr1	176833349	9.282	G	A	CAPZA2
	chr4	770570	9.252	A	G	PRSS12
	chr10	7988179	9.064	C	G	LOC106017391
LDH	chr18	7483362	10.410	A	G	ABL1
	chr8	28564827	9.637	A	G	LOC113844332
	chr18	7327690	9.550	G	A	NUP214
	chr28	4026847	9.273	G	A	KAT7
HBDH	chr18	7483362	10.354	A	G	ABL1
	chr8	28564827	10.236	A	G	LOC113844332
	chr28	4026847	9.134	G	A	KAT7
	chr1	3350683	9.032	G	A	CAB2
	chr15	11563909	8.981	C	T	TRAP1

#### II. GWAS for metabolism and protein-related traits

**Fig. 3:**
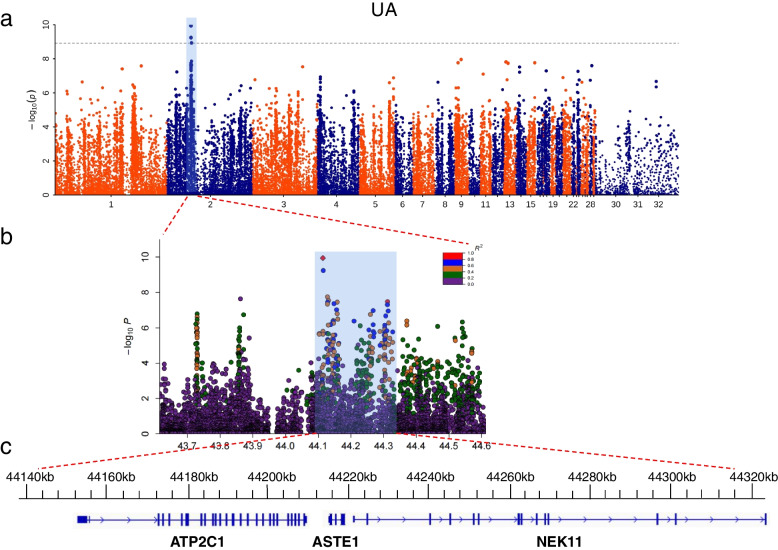
GWAS analysis of the UA. **a** Manhattan plot of UA. The gray horizontal dashed lines indicate the Bonferroni significance threshold of the GWAS (Correction threshold = 8.916). **b** Regional plots for the loci ranging from 43.6 to 44.6 Mbp associated with UA. **c** there are three genes (*ATP2C1*, *ASTE1*, *NEK11*) in the candidate region

**Fig. 4:**
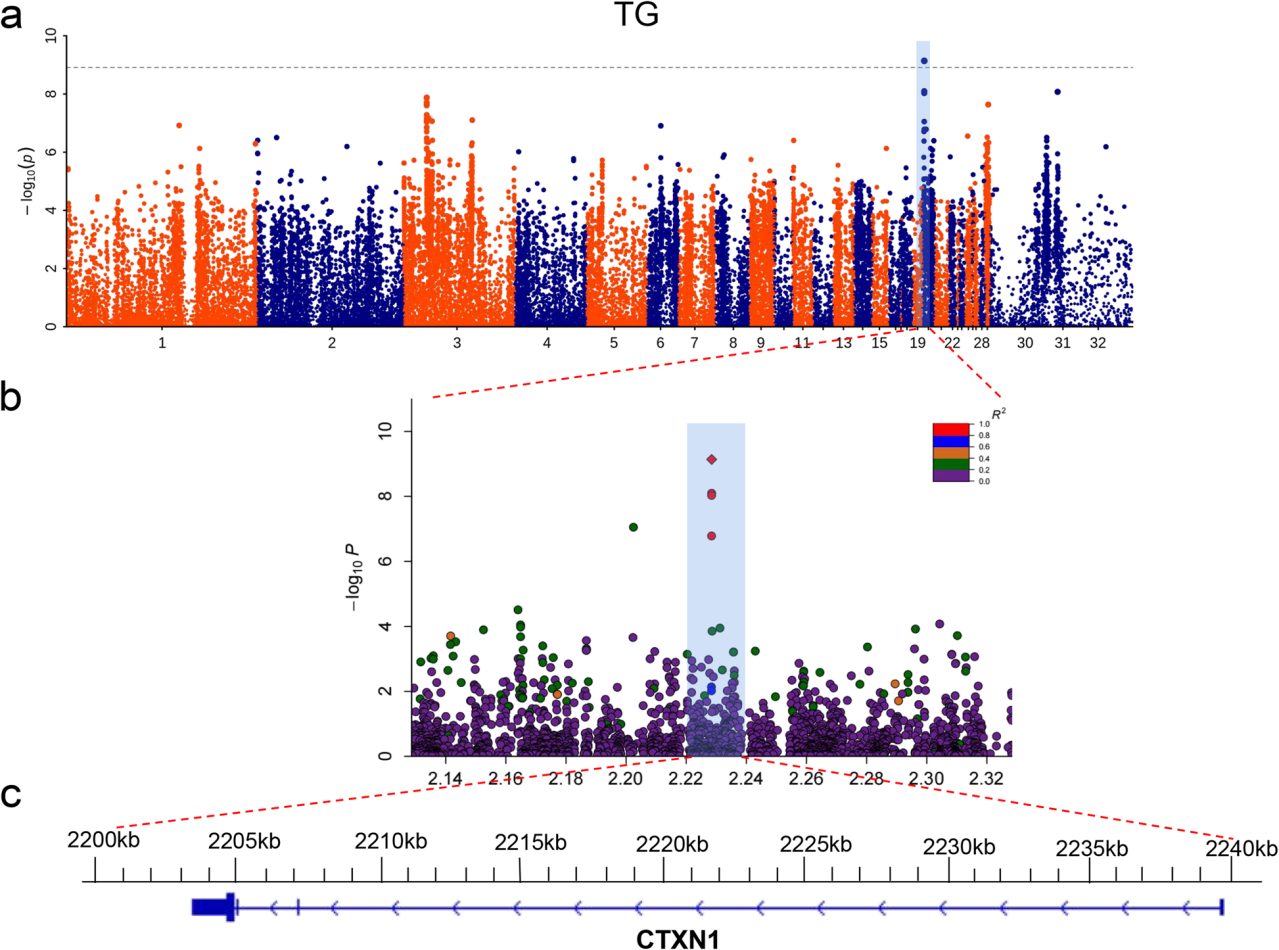
GWAS analysis of the TG. **a** Manhattan plot of TG. The gray horizontal dashed lines indicate the Bonferroni significance threshold of the GWAS (Correction threshold = 8.916). **b** Regional plots for the loci ranging from 2.13 to 2.33 Mbp associated with TG. **c**, there is only *CTXN1* in the candidate region

The Manhattan plot of UA only showed obvious signals on chromosome 2 (Fig. 3a). A total of 3 SNPs reached the significant threshold level. The significant SNPs were distributed in the 44113911 – 45103209 bp on chromosome 2. The regions harbored the candidate genes, including *ATP2C1* and *TMEM108* (Table 2). Then, we examined the leader SNP (Chr2: 44113911 bp) closely by calculating correlations between the SNPs within the QTL (Chr2:43.61–44.61 Mbp) surrounding the leader SNP on chromosome 2, and 56 SNPs were highly correlated (pairwise r^2^ > 0.6; Table S2 and Fig. 3b). In this range, we identified three candidate genes, including *ATP2C1*, *ASTE1*, *NEK11* (Fig. 3c). The results of TG only showed that 1 SNP (Chr20: 2228453 bp) reached the significance threshold level (Fig. 4a), and was distributed in the *CTXN1* on chromosome 20 (Table 2). Similarly, we calculated correlations between the SNPs within the QTL (Chr20: 1.73–2.73 Mbp) surrounding the leader SNP on chromosome 20, and 6 SNPs were highly correlated (pairwise r^2^ > 0.6; Table S3 and Fig. 4b). Only *CTXN1* (Fig. 4c) is near the leader SNP.

**Table 2:** The significant single nucleotide polymorphisms (SNPs) identified in genome-wide association studies for the metabolism and protein related traits

Trait	CHROM	POS	-log_10_(P)	REF	ALT	Close protein coding gene
UA	chr2	44113911	9.945	A	G	ATP2C1
	chr2	44115181	9.243	G	A	ATP2C1
	chr2	45103209	8.930	A	C	TMEM108
TG	chr20	2228453	9.138	C	A	CTXN1

### Candidate genes expression analysis

#### I. Candidate genes expression analysis for enzyme traits

**Fig. 5:**
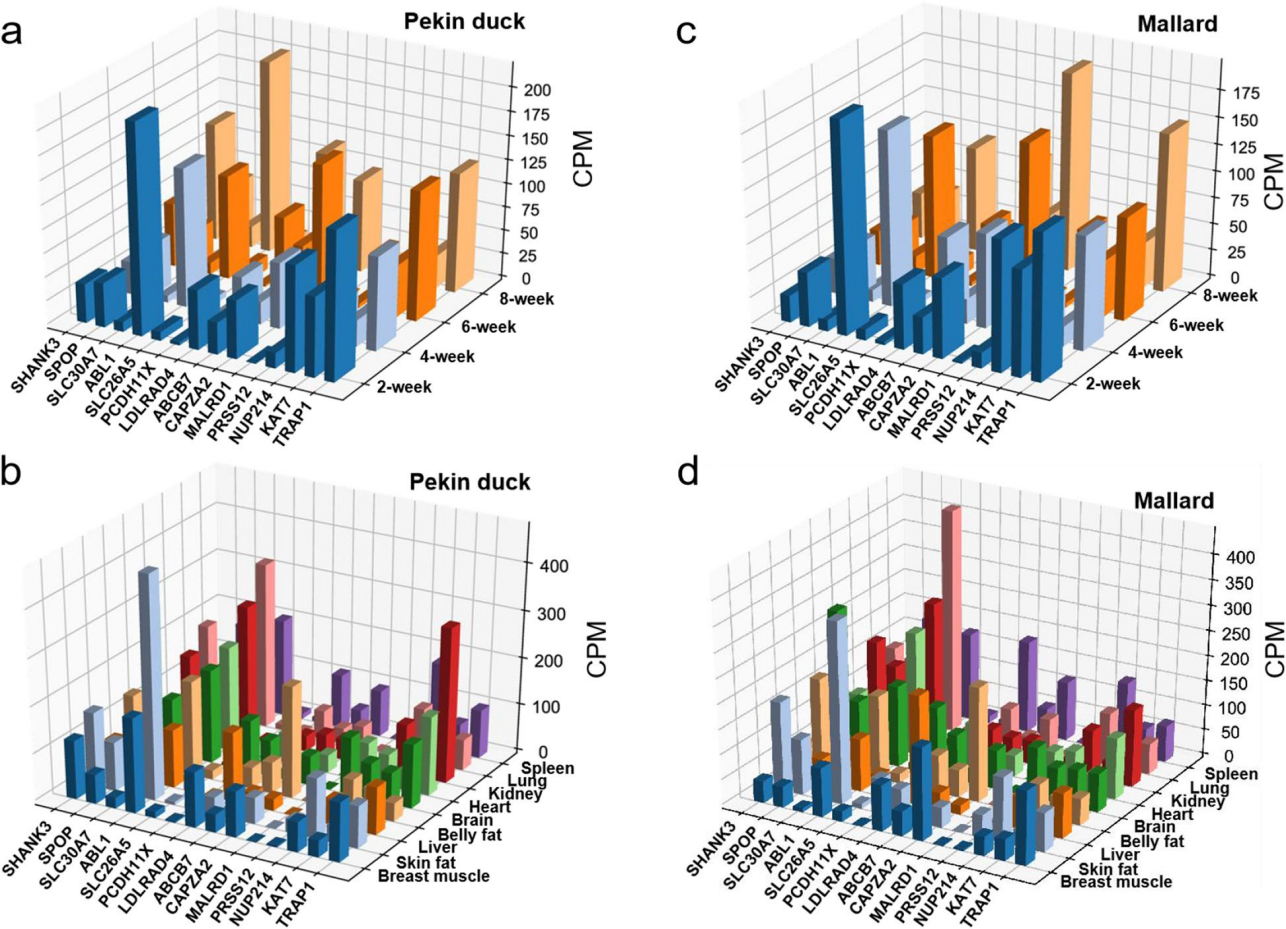
The expression level of candidate protein-coding genes related to enzyme traits was analyzed. **a**, **b**, Expression levels of putative candidate protein-coding genes in different stages of breast muscle and different tissues (Pekin duck). **c**, **d**, Expression levels of putative candidate protein-coding genes in different stages of breast muscle and different tissues (Mallard)

In animal breeding, serum biochemical parameters indirectly indicate health status and economic traits. To further determine the candidate genes for serum biochemical indicators, we compared the mRNA expression of candidate genes based on the transcriptome data in breast muscle tissues between Pekin ducks and Mallards during 2, 4, 6, and 8 weeks. Among the enzyme traits, 14 candidate protein-coding genes under the SNPs reached the significant threshold. Interestingly, *ABL1* was annotated in AST, LDH, and HBDH (Table 1) and had high expression in the breast muscle of Mallard and Pekin duck. At 2, 4, and 6 weeks, the expression level in the breast muscle of the Mallard was higher than that of the Pekin ducks, but at 8 weeks, the expression level in the breast muscle of the Pekin duck was higher than that of the Mallard. The *CAPZA2* gene was annotated in ALP and highly expressed in the breast muscle, and the expression level was higher in Mallard. Besides, *SHANK3*, *SPOP*, *LDLRAD4*, and *ABCB7* also have a relatively high expression level in the breast muscle (Fig. 5a and c).

In addition, we also analyzed the expression of candidate genes in different tissues (breast muscle, skin fat, liver, belly fat, brain, heart, kidney, lung, spleen). *ABL1* is widely and highly expressed in all tissues, especially in skin fat and lung, with the highest expression in the skin fat of Pekin duck and the highest expression in Mallard’s lung. *SHANK3*, *PCDH11X*, and *PRSS12* have the highest expression levels in the brain, *SPOP* expression level was highest in the brain, *LDLRAD4* was highly expressed in the liver and spleen, and the expression level in Mallard was higher than Pekin duck. *CAPZA2* has the highest expression level in belly fat (Fig. 5b and d).

#### II. Candidate genes expression analysis for metabolism and protein-related traits

**Fig. 6:**
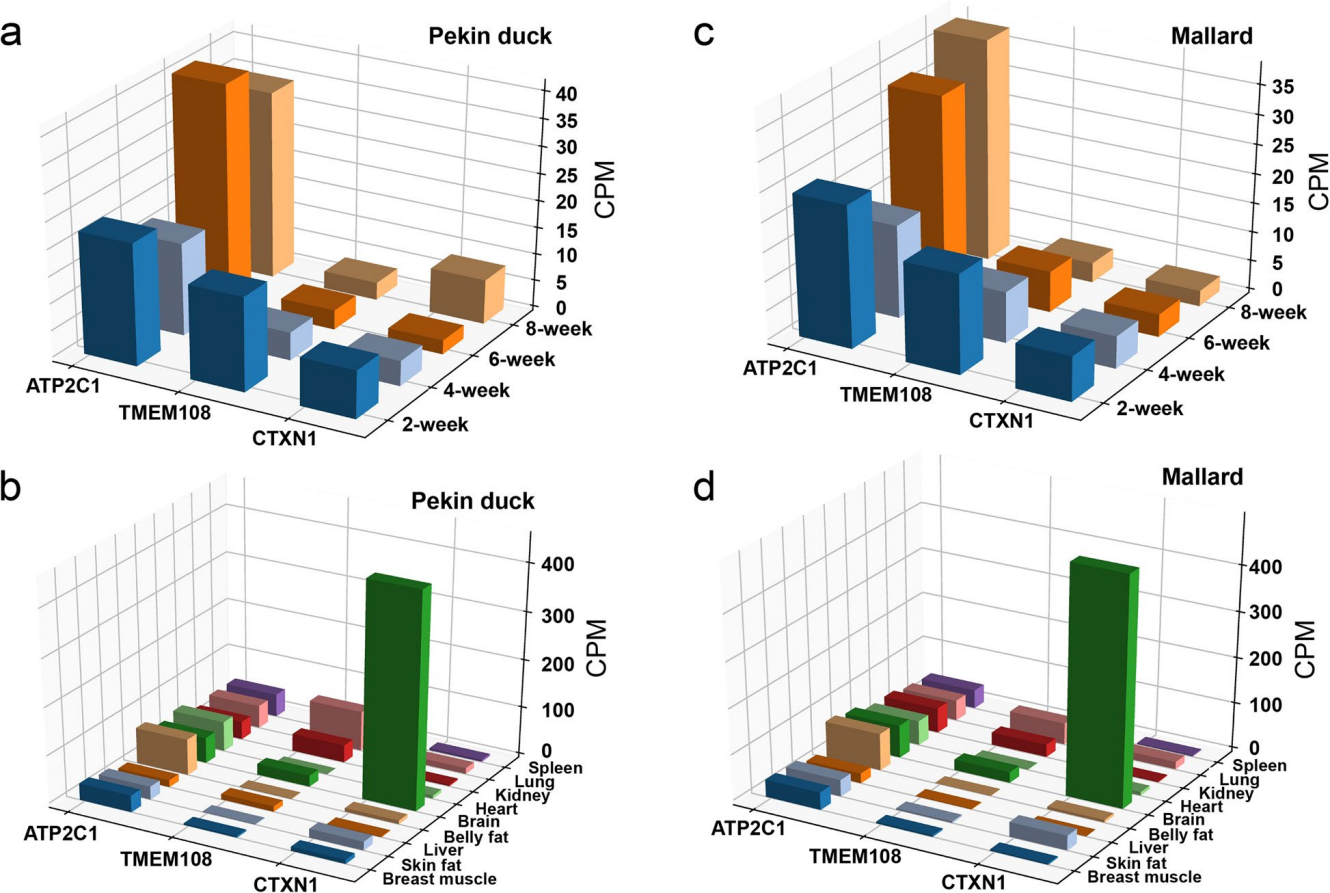
The expression level of candidate protein-coding genes related to metabolism and protein-related traits was analyzed. **a**, **b**, Expression levels of putative candidate protein-coding genes in different stages of breast muscle and different tissues (Pekin duck). **c**, **d**, Expression levels of putative candidate protein-coding genes in different stages of breast muscle and different tissues (Mallard)

The genes annotated by metabolism and protein-related traits only include *ATP2C1*, *TMEM108*, and *CTXN1*. Same as above, we analyzed the expression levels of candidate genes in the breast muscles of Pekin duck and Mallard at different growth stages (2, 4, and 6 weeks). *ATP2C1* was highly expressed in breast muscle, and *CTXN1* was less expressed in breast muscle. The expression level of *TMEM108* in the breast muscle of Pekin duck and Mallard at different stages showed a decreasing trend (Fig. 6a and c). Besides, the expression analysis of candidate genes in different tissues showed that *CTXN1* was especially highly expressed in the brain. *ATP2C1* was expressed in all tissues, with the highest in bally fat and the lowest in the liver. *TMEM108* was mainly expressed in the brain, kidney, and lung, and hardly expressed in other tissues (Fig. 6b and d).

### Candidate genes and functional annotation

**Fig. 7:**
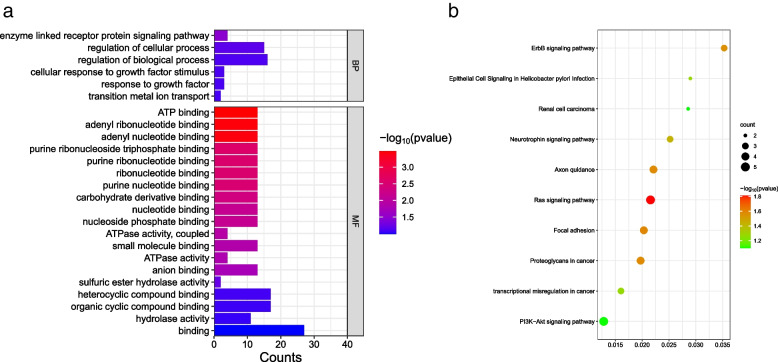
GO and KEGG enrichment analysis for 86 candidate genes. **a** GO enrichment analysis for 86 candidate genes. The x-axis indicates the number of genes for each GO term; the y-axis corresponds to the GO terms. The color of the bar represents the *P* value. **b** KEGG enrichment analysis for 86 candidate genes. The x-axis shows the gene ratio; the y-axis represents KEGG pathways. The dot color represents the *P* value, and the dot size represents the number of genes enriched in the reference pathway

The annotation of each important locus is considered as candidate gene. Moreover, the genes located within the high-LD region (r^2^ > 0.4 and − log_10_(*P*) < 4) neighboring the significant locus also remained. Finally, 86 putative protein-coding genes were used for GO and KEGG enrichment analysis (Table S4). Enzyme-linked receptor protein signaling pathway (GO:0,007,167) was the most significant GO term in the biological process. Within molecular function, ATP binding (GO:0,005,524) was the most dominant GO subcategory (Fig. 7b). Besides, another 10 GO terms also were significantly enriched, including adenyl ribonucleotide binding, adenyl nucleotide binding, purine ribonucleoside, etc. (Table S5). Five KEGG pathways were significantly enriched, including the ras signaling pathway, axon guidance, focal adhesion, proteoglycans in cancer, and the ErbB signaling pathway (Fig. 7b and Table S6).

## Discussion

Serum biochemical indicators can be used for the clinical detection of poultry's nutritional metabolism and growth performance. In this study, we measured the 18 serum parameters of ducks. The phenotypic correlation analysis showed that some serum biochemical indicators have high correlations. Like LDH and HBDH have high correlation, HBDH is an indirect reflection of LDH activity, and its activity changes parallel to the total LDH activity, the increase or decrease of LDH and HBDH remained concomitant, and serum parameters are mostly determined by biological genetic material [19], so we can analyze the genetic mechanism of these serum parameters at the genome level. This has aroused our interest in revealing the genetic determinants of duck serum biochemical indicators through genome-wide association study.

AST mainly exists in the mitochondria and cytoplasm of hepatocytes and is an essential enzyme for protein synthesis in hepatocytes. This enzyme is released into the blood when liver cells degenerate and necrosis or increase cell membrane permeability and are usually used to detect liver health as a clinical biomarker [20]. LDH and HBDH are usually used as one liver function indicators, and they are also used as cardiomyocyte markers in the clinical. Interestingly, our results showed that *ABL1*, *SPOP*, and *KAT7* were all annotated by these three enzymes (AST, LDH, HBDH) as candidate genes.

These genes can be proved to directly or indirectly affect the normal progress of liver or body life activities. It was reported that hepatocellular carcinoma (HCC) samples have increased levels of *ABL1* compared with nontumor liver tissues, and overexpress *ABL1* correlates with shorter survival times for patients. Knocking out *ABL1* or inhibiting its expression reduced HCC cells and slowed liver tumor growth in mice [21]. In addition, recent research has found that *ABL1* was associated with immune infiltration and the prognosis of HCC [22]. Gene expression analysis showed that the expression of the *ABL1* gene was most expressed in skin fat and lung, decreased in breast muscle at different stages, but increased at 8 W of Pekin duck. Studies have identified the critical role of *SPOP* in regulating proliferation and migration in liver cancer [23, 24]. Expression analysis showed that there was no differential expression of this gene. Bai found that miR-639 inhibits the proliferation and migration of human hepatocellular carcinoma cells through the KAT7/Wnt/β-Catenin Pathway, *KAT7* expression promotes cell proliferation and migration of human HCC cells in vitro [25]. Duck viral hepatitis (DVH) is one of the most serious infectious diseases in Pekin ducks [26]. Therefore, genetic analysis of serum biochemical indicators for evaluating liver function and screening candidate genes are highly important for duck quality breeding and liver performance determination.

ALP has essential physiological functions in the body, as a marker of osteoblasts maturation and an important indicator of bone metabolism [27, 28]. Our results showed that the candidate genes of ALP mainly include *ATRX*, *ALG13*, *CHRDL1*, and *AMMECR1*. Hypomorphic mutations of the *ATRX* could lead to skeletal deformities, and individuals with *ATRX* mutations show delayed bone age [29, 30]. A study has identified *ALG13* as a potential osteoporosis marker gene related to osteoclast activity and hypogonadal bone loss. CHRDL1 is a secreted glycoprotein, which can bind to BMPs family ligands and promote osteoblast differentiation in vivo [31]. Research in humans found that *AMMECR1* is potentially involved in cell cycle control and linked to a new syndrome with bone alterations [32].

The metabolism and protein-related traits included UA and TG. UA, the end product of purine metabolism is excreted predominantly by the proximal tubules. UA is a marker of kidney disease and is also associated with hypertension, gout, hyperuricemia, and some cardiovascular diseases [33, 34]. *PIK3R4* is the candidate gene for UA, which can cause ciliopathies and affect kidney function [35]. In human, *TMEM108* is a candidate gene associated with stroke by GWAS [36]. TG is an important indicator of heart health. In this study, there is only *CTXN1* in the candidate QTL region. However, there are few studies on this gene. Interestingly, this gene is highly expressed in the brain, then the details of the gene may require further study.

To gain insight into the function of 86 candidate genes, we performed GO and KEGG enrichment analyses. GO terms with major enrichment of candidate genes, including the enzyme linked-receptor protein signaling pathway, ATP binding, and enzymatic activity. Therefore, we speculate that candidate genes related to blood biochemical indicators play an essential role in biological processes such as energy metabolism. ALP in the body plays a crucial role in cell cycle, growth, apoptosis and signal transduction pathways, also is a marker of osteoblast maturation and an important indicator of bone metabolism [27, 37]. As a candidate gene related to ALP, *CAPZA2* highly expressed in the breast muscle and belly fat, which enriched in enzyme-linked receptor protein signaling pathway, regulation of cellular process and biological process. Researchers found that a de novo inframe deletion variant in *CAPZA2* tentacle domain with global developmental delay and skeletal malformation of head [38]. Therefore, *CAPZA2* could be further studied as an essential gene. In addition, ErbB signaling pathway focused by us, the candidate gene *ABL1* of AST, LDH and HBDH is enriched in this pathway, and the pathway plays a key role in the development of many cancers and the immune response [39], *ABL1* also is also involved in the carcinogenesis and immune process.

## Conclusion

In summary, we detected 18 serum biochemical indicators and analyzed them by GWAS in this study. We found 6 serum parameter phenotypic indicators showing significant signals by GWAS analysis. Expression analysis of 14 putative candidate protein-coding genes related to enzyme traits and 3 candidate protein-coding genes related to metabolism and protein-related traits were performed. The candidate genes and SNPs found in this study may contribute to the future research of serum biomarkers and provide a reference for the early breeding of ducks.

## Methods

### Experimental population and sample preparation

The Mallard × Pekin F_2_ resource population used in this study was established by the Key Laboratory of Animal (Poultry) Genetics Breeding and Reproduction. The F_2_ segregating population description refers to Zhou et al. [40]. In the orthogonal cross, 10 ♂ Pekin ducks × 100 ♀ Mallard ducks were selected as parents. In the reciprocal cross, 4 ♂ Mallard ducks × 40 ♀ Pekin ducks were selected as parents, and nearly 2,000 F_2_ ducks were finally generated. All ducks had free access to feed and water and were managed in the same environment. In this study, 320 ducks randomly sampled.

### Biochemical indicators’ measurements

Total 320 blood samples were collected from the wing vein of ducks and stored at 4 °C until centrifuged at 3000 rpm for 10 min to obtain serum. The levels of plasma parameters were measured using an automatic analyzer (Hitachi 7080, Japan) with a commercial kit (Maccura, China), including alanine aminotransferase (ALT), aspartate aminotransferase (AST), total protein (TP), albumin (ALB), total bilirubin (TBIL), alkaline phosphatase (ALP), glucose (GLU), urea nitrogen (BUN), uric acid (UA), phosphorus(P), total cholesterol (CHO), triglyceride (TG), high-density lipoprotein cholesterol (HDL-C), low-density lipoprotein cholesterol (LDL-C), lipoprotein(a) (LP(a)), creatine kinase (CK), lactate dehydrogenase (LDH), α- Hydroxybutyrate dehydrogenase (HBDH).

### DNA isolation and sequencing

Genomic DNA was extracted from the blood using the standard phenol/chloroform extraction method. Nanodrop and agarose gel electrophoresis estimated the quality of DNA. Generate two paired-end libraries using standard procedures according to the manufacturer's protocol (Illumina, USA). The average insert size is 500 bp, and the read length is 150 bp. Ultimately, these libraries were sequenced on the Illumina® Hiseq X-Ten platform.

### Variant detection and genotyping

The 2 × 150-bp paired-end reads were mapped to the Pekin duck reference genome (IASCAAS_Peking Duck_PBH1.5, GCF_003850225.1). After that, SNPs calling was performed using the GATK (version 3.5.0) HaplotypeCaller tool [41] with the following cut-off values: QUAL < 100.0, MQ < 40.0, QD < 2.0, SOR > 3.0, FS > 60.0, ReadPosRankSum < -8.0, and MQRankSum < -12.5. The output was further filtered using VCFtools (Version 0.1.15) [42], and the criteria were as follows: Only SNPs with minor allele frequencies above 0.05 and maximum allele frequencies below 0.99 were retained, and the maximum missing rate was set at < 0.1 and SNPs had to have only two alleles. After filtering, 320 ducks from an F_2_ segregating population mated by Pekin duck and Mallard were genotyped, and 8,234,067 SNPs were prepared for subsequent analysis.

### GWAS

GWAS was performed on the phenotype Indicators with the mixed linear model program EMMAX [43]. Population structure and cryptic relationships were considered to minimize false positives and increase statistical power. The first three principal component values (PCA eigenvectors) are set as a fixed effect in the mixed model to correct population stratification [44]. The Random effect was the phylogenetic matrix estimated by all genome-wide SNPs. We defined the whole-genome significance cutoff as the Bonferroni threshold, 0.01/Total SNPs (− log 10 (*P*) = 8.916). The linear model is as follows:

$$\mathbf{y}=\mathbf{X}{\varvec{\upalpha}}+\mathbf{Z}{\varvec{\upbeta}}+\mathbf{W}{\varvec{\upmu}}+\mathbf{e}$$

where **y** is the vector of phenotypic values of serum biochemical indicators, **Xα** is the fixed effects; **Zβ** represents the effect of SNP, and **β** represents allele substitution effect; **Wµ** represents random animal effects with a variance–covariance structure based on the kinship matrix estimated using whole-genome SNP genotypes, and **e** is random residuals for perimysial thickness data.

### Total RNA isolation and construction of RNA-seq libraries

Pekin duck and Mallard collected multiple tissues (breast muscle, skin fat, liver, belly fat, brain, heart, kidney, lung, spleen). In detail, 2 W, 4 W, 6 W, and 8 W, breast muscles of 3 Pekin ducks and 3 Mallards were collected, respectively. Other tissues were collected at 8 W. The total RNA was isolated with Trizol reagent (Takara), and then the integrity and concentration were estimated using a NanoDrop spectrophotometer (Thermo Fisher Scientific, USA), and verified using the agarose gel method. Only qualified samples were purified for RNA-seq library construction. The libraries meeting the quality criteria were sequenced using the Illumina Hiseq 4000 platform, which generated paired-end reads of 150 bp. RNA-seq paired-end reads were mapped to the Pekin duck reference genome (GCA_003850225.1) using TopHat version 2.0.11 software [45]. Subsequently, read counts per million (CPM) of the genes were obtained by running htseq-count [46]. CPM-mapped sequence reads for each gene were calculated by edgeR version 3.20.9 package, where CPM represents the gene expression level [47].

### Candidate genes and functional annotation

To identify the positional candidate genes that are potentially associated with serum indicators, the genes located within the high-LD region (r^2^ > 0.4 and − log_10_(*P*) < 4) neighboring the significant locus also remained [48]. These regions were then referenced against the duck reference genome (IASCAAS_Peking Duck_PBH1.5, GCF_003850225.1) to find genes located in the vicinity of the significant SNPs. The candidate genes were performed GO enrichment analysis and KEGG enrichment analysis using the DAVID website (DAVID: Functional Annotation Tools (ncifcrf.gov)).

## Supplementary Information

**Additional file 1: Figure S1.** Quantile-quantile (QQ) plot on Serum AST, ALP, LDH, HBDH, UA and TG.

**Additional file 2: Figure S2.** Manhattan and quantile-quantile (QQ) plot on Serum ALT, TP, ALB, TBIL, GLU, and BUN.

**Additional file 3: Figure S3.** Manhattan and quantile-quantile (QQ) plot on Serum P, CHO, HDL-C, LDL-C, LP(a) and CK.

**Additional file 4: Table S1.** Determination results of 18 blood biochemical indicators.

**Additional file 5: Table S2.** SNPs with a pairwise r2 > 0.6 with the leader SNP at chr2: 44113911 bp.

**Additional file 6: Table S3.** SNPs with a pairwise r2 > 0.6 with the leader SNP at chr20: 2228453 bp.

**Additional file 7: Table S4.** Candidate genes for enrichment analysis. The putative candidate genes, including the genes annotated on potential candidate SNPs (top 10) and genes located in the genomic region (r2 > 0.4 and -log10(P) < 4). A total of 85 unique potential candidate genes were identified for KEGG enrichment analysis.

**Additional file 8: Table S5.** The result of GO enrichment analysis.

**Additional file 9: Table S6**. The result of the KEGG enrichment analysis.

## Data Availability

In this study, all sequences supporting the conclusions are deposited at the Sequence Read Archive (https://www.ncbi.nlm.nih.gov/sra) with the accession number PRJNA471401 and PRJNA450892. The genome assembly, sequence data, and SNP information were deposited in BIG Data Center (http://bigd.big.ac.cn/) with the accession numbers PRJCA000651, PRJCA000647, and GVM000015. The RNA-Seq datasets used in this study are available at BIG Data Center (http://bigd.big.ac.cn/) with the accession number PRJCA001307. For Other data supporting the results of this study, see the supplementary file.

## Abbreviations

LDH: Lactate dehydrogenase
HBDH: α-Hydroxybutyrate dehydrogenase
AST: Aspartate aminotransferase
ALT: Alanine aminotransferase
TP: Total protein
ALB: Albumin
HDL-C: High-density lipoprotein cholesterol
LDL-C: Low density lipoprotein cholesterol
CHO: Cholesterol
TG: Triglyceride
CK: Creatine kinase
GLU: Glucose
TBIL: Total bilirubin
BUN: Urea nitrogen
P: Phosphorus
GWAS: Genome-wide association study
UA: Uric acid
LD: Linkage disequilibrium
GO: Gene Ontology
KEGG: Kyoto Encyclopedia of Gene and Genomes database
PSE: Pale, soft, and exudative meat
QTL: Quantitative trait loci
LP(a): Lipoproteins(a)
QQ plot: Quantile–Quantile plot
SNP: Single nucleotide polymorphism
HCC: Hepatocellular carcinoma
