# Supplementary figures and images for: Genome-wide association study reveals the genetic determinism of serum biochemical indicators in ducks
Source: BMC Genomics. 2022 Dec 27;23:856. doi: 10.1186/s12864-022-09080-9 (PMC9795613; doi:10.1186/s12864-022-09080-9)

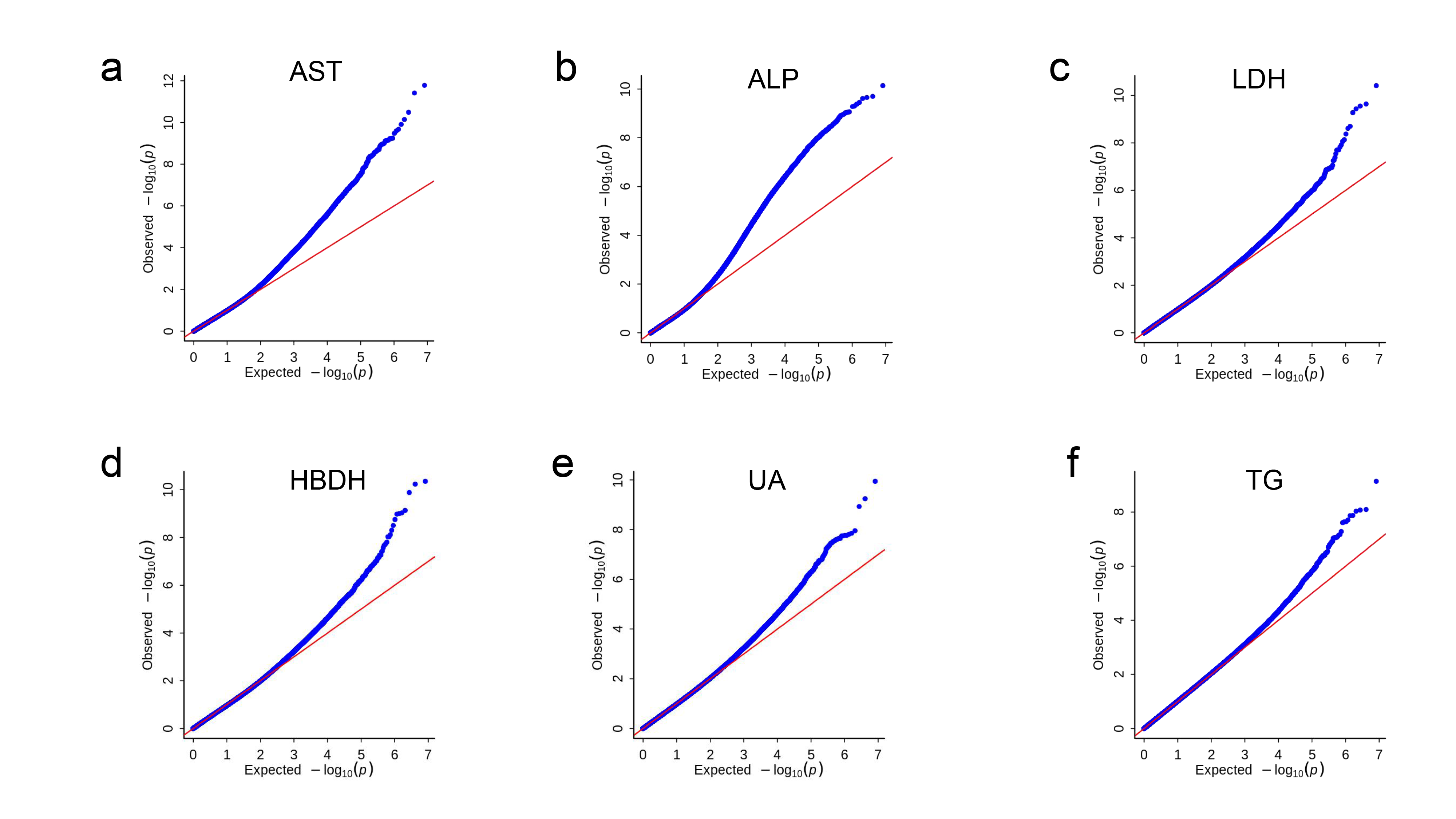

Supplement: Supplementary file 1 — Additional file 1: Figure S1. Quantile-quantile (QQ) plot on Serum AST, ALP, LDH, HBDH, UA and TG. [file 12864_2022_9080_MOESM1_ESM.tif]

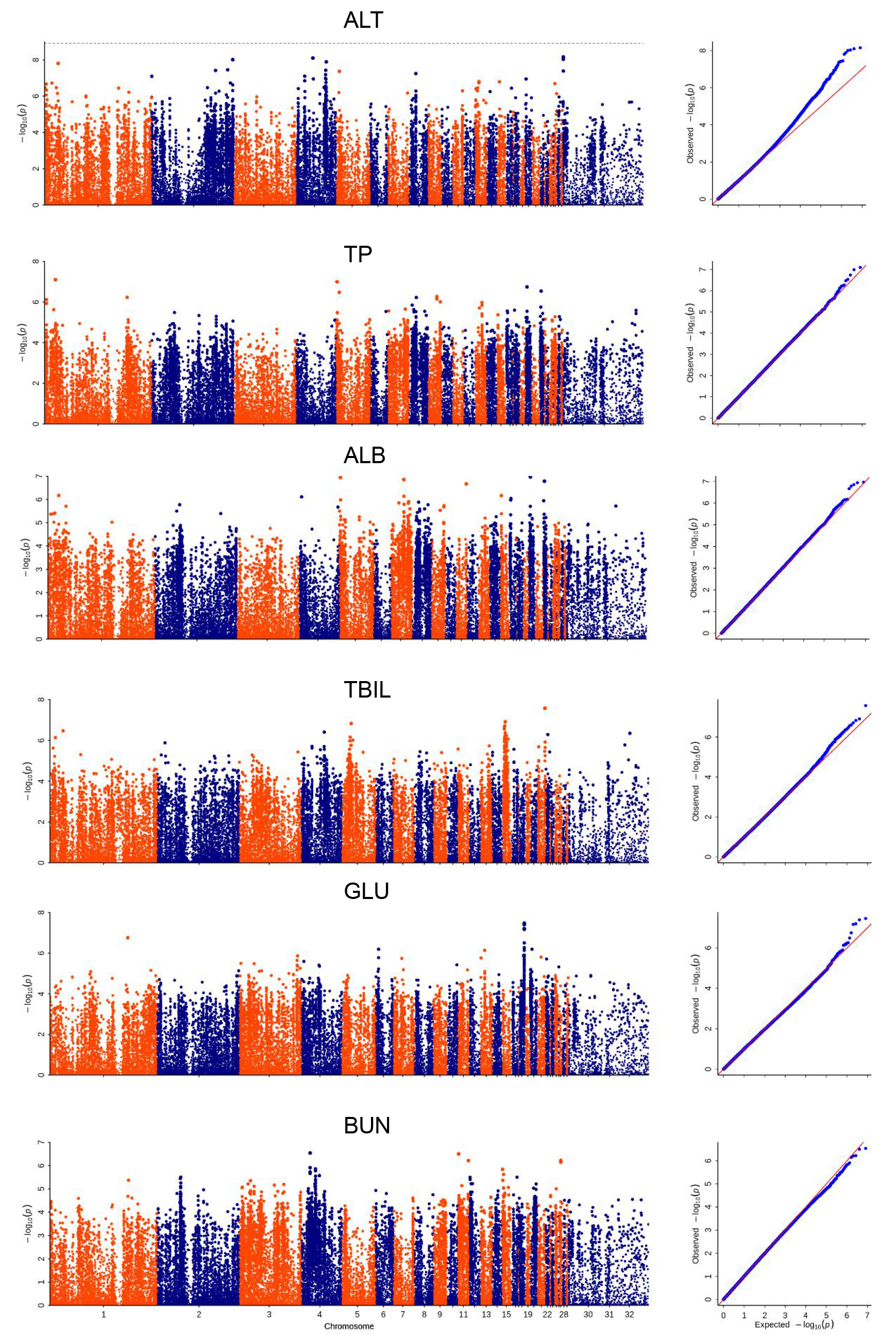

Supplement: Supplementary file 2 — Additional file 2: Figure S2. Manhattan and quantile-quantile (QQ) plot on Serum ALT, TP, ALB, TBIL, GLU, and BUN. [file 12864_2022_9080_MOESM2_ESM.tif]

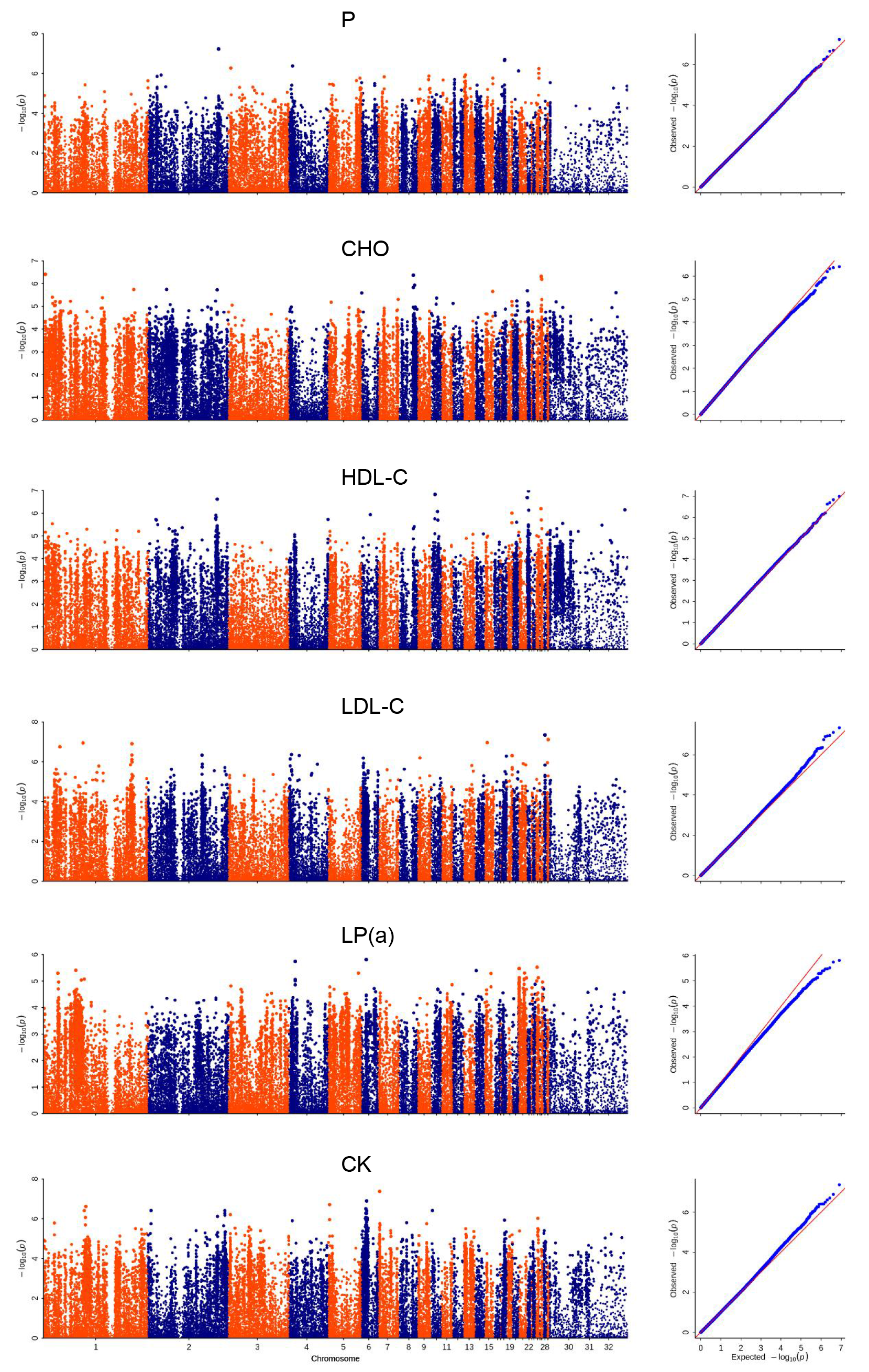

Supplement: Supplementary file 3 — Additional file 3: Figure S3. Manhattan and quantile-quantile (QQ) plot on Serum P, CHO, HDL-C, LDL-C, LP(a) and CK. [file 12864_2022_9080_MOESM3_ESM.tif]
