# Supplementary material for: Genome-wide association study reveals the genetic determinism of serum biochemical indicators in ducks
Source: BMC Genomics. 2022 Dec 27;23:856. doi: 10.1186/s12864-022-09080-9 (PMC9795613; doi:10.1186/s12864-022-09080-9)
Supplement: Supplementary file 4 — Additional file 4: Table S1. Determination results of 18 blood biochemical indicators. [file 12864_2022_9080_MOESM4_ESM.docx]

| Trait | Mean±SD | CV |
| --- | --- | --- |
| ALT | 35.95±9.77 | 0.27 |
| AST | 22.23±13.19 | 0.59 |
| TP | 32.59±3.49 | 0.11 |
| ALB | 13.51±1.32 | 0.1 |
| TBIL | 26.73±4.26 | 0.16 |
| ALP | 9.68±11.83 | 1.22 |
| GLU | 8.45±0.92 | 0.11 |
| BUN | 0.4±0.08 | 0.21 |
| UA | 155.73±54.02 | 0.35 |
| P | 1.98±0.21 | 0.11 |
| CHO | 6.21±0.82 | 0.13 |
| TG | 0.83±0.19 | 0.23 |
| HDL-C | 3.5±0.49 | 0.14 |
| LDL-C | 1.74±0.39 | 0.23 |
| LP(a) | 7.3±4.01 | 0.55 |
| CK | 850.06±252.8 | 0.3 |
| LDH | 456.65±245.73 | 0.54 |
| HBDH | 387.55±293.22 | 0.76 |

**Table S1 Determination results of 18 blood biochemical indicators**
