# Supplementary material for: Genome-wide association study reveals the genetic determinism of serum biochemical indicators in ducks
Source: BMC Genomics. 2022 Dec 27;23:856. doi: 10.1186/s12864-022-09080-9 (PMC9795613; doi:10.1186/s12864-022-09080-9)
Supplement: Supplementary file 5 — Additional file 5: Table S2. SNPs with a pairwise r2 > 0.6 with the leader SNP at chr2: 44113911 bp. [file 12864_2022_9080_MOESM5_ESM.docx]

| **Table S2 SNPs with a pairwise r^2^ > 0.6 with the leader SNP at chr2: 44113911 bp** | | | | |
| --- | --- | --- | --- | --- |
| CHR_B | BP_B | SNP_B | r^2^ |  |
| 2 | 44113911 | snp1890547 | 1 |  |
| 2 | 44311802 | snp1892761 | 0.842331 |  |
| 2 | 44127711 | snp1890758 | 0.759091 |  |
| 2 | 44128629 | snp1890770 | 0.757047 |  |
| 2 | 44311578 | snp1892755 | 0.744651 |  |
| 2 | 44163083 | snp1891210 | 0.74174 |  |
| 2 | 44149645 | snp1891021 | 0.715361 |  |
| 2 | 44111754 | snp1890490 | 0.714447 |  |
| 2 | 44147804 | snp1890998 | 0.702019 |  |
| 2 | 44254765 | snp1892382 | 0.69228 |  |
| 2 | 44140841 | snp1890915 | 0.691612 |  |
| 2 | 44288932 | snp1892564 | 0.682823 |  |
| 2 | 44302526 | snp1892668 | 0.676844 |  |
| 2 | 44154401 | snp1891103 | 0.676499 |  |
| 2 | 44270958 | snp1892502 | 0.676453 |  |
| 2 | 44151284 | snp1891030 | 0.672418 |  |
| 2 | 44145863 | snp1890984 | 0.668319 |  |
| 2 | 44303829 | snp1892683 | 0.667535 |  |
| 2 | 44159197 | snp1891150 | 0.66647 |  |
| 2 | 44128836 | snp1890775 | 0.66497 |  |
| 2 | 44145582 | snp1890980 | 0.662999 |  |
| 2 | 44327647 | snp1893015 | 0.661658 |  |
| 2 | 44254712 | snp1892381 | 0.656232 |  |
| 2 | 44302530 | snp1892669 | 0.655897 |  |
| 2 | 44302531 | snp1892670 | 0.655897 |  |
| 2 | 44125212 | snp1890742 | 0.651891 |  |
| 2 | 44125216 | snp1890743 | 0.651891 |  |
| 2 | 44125224 | snp1890744 | 0.651891 |  |
| 2 | 44128835 | snp1890774 | 0.648131 |  |
| 2 | 44129304 | snp1890786 | 0.643992 |  |
| 2 | 44309864 | snp1892719 | 0.640684 |  |
| 2 | 44102672 | snp1890344 | 0.640269 |  |
| 2 | 44163024 | snp1891206 | 0.640164 |  |
| 2 | 44304300 | snp1892687 | 0.638337 |  |
| 2 | 44302953 | snp1892678 | 0.63727 |  |
| 2 | 44209340 | snp1891779 | 0.636857 |  |
| 2 | 44269384 | snp1892495 | 0.63459 |  |
| 2 | 44135976 | snp1890867 | 0.634515 |  |
| 2 | 44146040 | snp1890985 | 0.631015 |  |
| 2 | 44304904 | snp1892691 | 0.627212 |  |
| 2 | 44137918 | snp1890891 | 0.625038 |  |
| 2 | 44301745 | snp1892663 | 0.623293 |  |
| 2 | 44267880 | snp1892489 | 0.623226 |  |
| 2 | 44313740 | snp1892787 | 0.622292 |  |
| 2 | 44128740 | snp1890772 | 0.616132 |  |
| 2 | 44306411 | snp1892703 | 0.616024 |  |
| 2 | 44159240 | snp1891151 | 0.615807 |  |
| 2 | 44303103 | snp1892680 | 0.614892 |  |
| 2 | 44302936 | snp1892677 | 0.613071 |  |
| 2 | 44159053 | snp1891148 | 0.612261 |  |
| 2 | 44129293 | snp1890785 | 0.611556 |  |
| 2 | 44155796 | snp1891120 | 0.611005 |  |
| 2 | 44115181 | snp1890590 | 0.610214 |  |
| 2 | 44268624 | snp1892491 | 0.607576 |  |
| 2 | 44302617 | snp1892671 | 0.602023 |  |
| 2 | 44267905 | snp1892490 | 0.600177 |  |
