# Supplementary material for: Genome-wide association study reveals the genetic determinism of serum biochemical indicators in ducks
Source: BMC Genomics. 2022 Dec 27;23:856. doi: 10.1186/s12864-022-09080-9 (PMC9795613; doi:10.1186/s12864-022-09080-9)
Supplement: Supplementary file 6 — Additional file 6: Table S3. SNPs with a pairwise r2 > 0.6 with the leader SNP at chr20: 2228453 bp. [file 12864_2022_9080_MOESM6_ESM.docx]

**Table S3 SNPs with a pairwise r^2^ > 0.6 with the leader SNP at chr20: 2228453 bp**

| CHR_B | BP_B | SNP_B | r^2^ |
| --- | --- | --- | --- |
| 20 | 2228453 | snp7334131 | 1 |
| 20 | 2228454 | snp7334132 | 0.980369 |
| 20 | 2228430 | snp7334128 | 0.915113 |
| 20 | 2228419 | snp7334127 | 0.894254 |
| 20 | 2228374 | snp7334125 | 0.642727 |
| 20 | 2228389 | snp7334126 | 0.609414 |
