# Supplementary material for: Genome-wide association study reveals the genetic determinism of serum biochemical indicators in ducks
Source: BMC Genomics. 2022 Dec 27;23:856. doi: 10.1186/s12864-022-09080-9 (PMC9795613; doi:10.1186/s12864-022-09080-9)
Supplement: Supplementary file 8 — Additional file 8: Table S5. The result of GO enrichment analysis. [file 12864_2022_9080_MOESM8_ESM.docx]

| Term | Count | *P*-value | group |
| --- | --- | --- | --- |
| enzyme linked receptor protein signaling pathway | 4 | 0.032357648 | BP |
| regulation of cellular process | 15 | 0.0641749 | BP |
| regulation of biological process | 16 | 0.07362879 | BP |
| cellular response to growth factor stimulus | 3 | 0.074568702 | BP |
| response to growth factor | 3 | 0.075936359 | BP |
| transition metal ion transport | 2 | 0.078152971 | BP |
| ATP binding | 13 | 3.32E-04 | MF |
| adenyl ribonucleotide binding | 13 | 3.61E-04 | MF |
| adenyl nucleotide binding | 13 | 3.76E-04 | MF |
| purine ribonucleoside triphosphate binding | 13 | 0.0025728 | MF |
| purine ribonucleotide binding | 13 | 0.002775768 | MF |
| ribonucleotide binding | 13 | 0.002969642 | MF |
| purine nucleotide binding | 13 | 0.003082114 | MF |
| carbohydrate derivative binding | 13 | 0.004475513 | MF |
| nucleoside phosphate binding | 13 | 0.006860487 | MF |
| nucleotide binding | 13 | 0.006860487 | MF |
| ATPase activity, coupled | 4 | 0.011562811 | MF |
| small molecule binding | 13 | 0.013211169 | MF |
| ATPase activity | 4 | 0.014399489 | MF |
| anion binding | 13 | 0.017641565 | MF |
| sulfuric ester hydrolase activity | 2 | 0.056682298 | MF |
| heterocyclic compound binding | 17 | 0.079180758 | MF |
| organic cyclic compound binding | 17 | 0.083176013 | MF |
| hydrolase activity | 11 | 0.08592305 | MF |
| binding | 27 | 0.099685073 | MF |

**Table S5 The result of GO enrichment analysis**
