# Supplementary material for: Genome-wide association study reveals the genetic determinism of serum biochemical indicators in ducks
Source: BMC Genomics. 2022 Dec 27;23:856. doi: 10.1186/s12864-022-09080-9 (PMC9795613; doi:10.1186/s12864-022-09080-9)
Supplement: Supplementary file 9 — Additional file 9: Table S6. The result of the KEGG enrichment analysis. [file 12864_2022_9080_MOESM9_ESM.docx]

**Table S6 The result of KEGG enrichment analysis**

| pathway | count | rich_factor | pvalue |
| --- | --- | --- | --- |
| ErbB signaling pathway | 3 | 0.035294118 | 0.0275 |
| Renal cell carcinoma | 2 | 0.028571429 | 0.0785 |
| Epithelial Cell Signaling in Helicobacter pylori Infection | 2 | 0.028985507 | 0.0531 |
| Neurotrophin signaling pathway | 3 | 0.025210084 | 0.0369 |
| Axon quidance | 4 | 0.022099448 | 0.0266 |
| Ras signaling pathway | 5 | 0.021551724 | 0.0154 |
| Focal adhesion | 4 | 0.020304569 | 0.0258 |
| Proteoglycans in cancer | 4 | 0.019704433 | 0.0265 |
| transcriptional misregulation in cancer | 3 | 0.016042781 | 0.0536 |
| PI3K-Akt signaling pathway | 5 | 0.012820513 | 0.0782 |
